# Is evidence-informed urban health planning a myth or reality? Lessons from a qualitative assessment in three Asian cities

**DOI:** 10.1093/heapol/czz097

**Published:** 2019-10-11

**Authors:** Tolib Mirzoev, Ak Narayan Poudel, Stefanie Gissing, Thi Thuy Duong Doan, Tarana Ferdous, Shophika Regmi, Minh Duc Duong, Sushil Baral, Obindra Chand, Rumana Huque, Van Minh Hoang, Helen Elsey

**Affiliations:** 1 University of Leeds, Leeds Institute of Health Sciences, Nuffield Centre for International Health and Development, 10.31b, Worsley Building, Clarendon Way, Leeds LS2 9NL, UK; 2 Hanoi University of Public Health, 1A Duc Thang, North Tu Liem, Hanoi, Vietnam; 3 ARK Foundation, C4, House 6, Road 109, Gulshan 2, Dhaka-1212, Bangladesh; 4 HERD International, PO Box 24144, Thapathali Height 11, Kathmandu, Nepal 44600

**Keywords:** Urban health, planning, evidence, data

## Abstract

City governments are well-positioned to effectively address urban health challenges in the context of rapid urbanization in Asia. They require good quality and timely evidence to inform their planning decisions. In this article, we report our analyses of degree of data-informed urban health planning from three Asian cities: Dhaka, Hanoi and Pokhara. Our theoretical framework stems from conceptualizations of evidence-informed policymaking, health planning and policy analysis, and includes: (1) key actors, (2) approaches to developing and implementing urban health plans, (3) characteristics of the data itself. We collected qualitative data between August 2017 and October 2018 using: in-depth interviews with key actors, document review and observations of planning events. Framework approach guided the data analysis. Health is one of competing priorities with multiple plans being produced within each city, using combinations of top-down, bottom-up and fragmented planning approaches. Mostly data from government information systems are used, which were perceived as good quality though often omits the urban poor and migrants. Key common influences on data use include constrained resources and limitations of current planning approaches, alongside data duplication and limited co-ordination within Dhaka’s pluralistic system, limited opportunities for data use in Hanoi and inadequate and incomplete data in Pokhara. City governments have the potential to act as a hub for multi-sectoral planning. Our results highlight the tensions this brings, with health receiving less attention than other sector priorities. A key emerging issue is that data on the most marginalized urban poor and migrants are largely unavailable. Feasible improvements to evidence-informed urban health planning include increasing availability and quality of data particularly on the urban poor, aligning different planning processes, introducing clearer mechanisms for data use, working within the current systemic opportunities and enhancing participation of local communities in urban health planning.

**Key Messages**

- City municipalities are well-positioned to effectively address urban health issues with their inter-sectoral mandate, therefore, requiring appropriate data to inform planning decisions. This article reports the extent of data-informed urban health planning in three Asian cities: Dhaka (Bangladesh), Hanoi (Vietnam) and Pokhara (Nepal).
- Multiple plans are produced within each context, using combinations of primarily top-down, bottom-up and fragmented planning approaches. Mainstream information systems produce health-related data though there is insufficient detail on urban poor and migrant populations.
- A clear difference exists between the intended and actual data use in urban health planning. Key common contextual influences explaining limited data use include limited resources and limitations of current planning approaches within each context.
- Feasible improvements to the evidence-informed nature of urban health planning require alignment of planning and data processes and taking advantage of the multi-sectoral structural contexts and pluralistic organizational environments.

## Introduction

Rapid urbanization in Asia poses a challenge for health systems to effectively address health needs of urban populations. Yet, urban health has received insufficient global and national attention (Stephens, 1990; Rydin *et al.*, 2012; Shawar and Crane, 2017). The urban advantage has been described as worthy of encouragement (Rydin *et al.*, 2012), though in reality urban health inequalities are substantial and rising, particularly disadvantaging migrant populations and the urban poor (Adelekan, 2010; Daniel *et al.*, 2013; Shawar and Crane, 2017; Nwameme *et al.*, 2018). City governments are well-positioned to effectively respond to this challenging and dynamic situations in many low- and middle-income countries (LMICs), given their relative autonomy, inter-sectoral nature and mandate to provide healthcare (Rydin *et al.*, 2012; Barton and Grant, 2013; Avelino *et al.*, 2014). However, the academic and policy work to understand how city governments plan their responses to urban health issues and the extent to which their planning decisions are informed by available evidence is limited.

Effective responses to health needs require timely and good-quality evidence (Lavis *et al.*, 2009; El-Jardali *et al.*, 2012; Ward *et al.*, 2012; Mirzoev *et al.*, 2013; 2017). A substantial literature helps to explain, assess and strengthen the role of evidence in health policymaking. It includes variety of approaches, models and processes of exchange between the key actors including key contextual influences on evidence-informed health policymaking (Dobrow *et al.*, 2006; Lavis *et al.*, 2009; El-Jardali *et al.*, 2012; Ward *et al.*, 2012; Koon *et al.*, 2013; Mirzoev *et al.*, 2013, 2017). Many scholars primarily equated evidence with research resulting in hierarchies of evidence by its methodological rigour (Lavis *et al.*, 2009; Grimshaw *et al.*, 2012; Lewin *et al.*, 2012). However, other conceptualizations included multiple forms of data and informal evidence types such as actors’ experiences (Rychetnik *et al.*, 2004; Shaxson, 2005; Mirzoev *et al.*, 2013). Nonetheless, most studies explored the role of research evidence in health policymaking with less emphases on the role of data from regular government information systems.

Frameworks for health planning processes range from detailed six-step processes comprising initial situational analysis, priority-setting, option appraisal, programming when a plan is formalized, implementation and monitoring, and evaluation (Green, 2007), through to simpler two-step ones which distinguish development and implementation but also highlight required capacity to plan and key influences on health planning (Wickremasinghe *et al.*, 2016; Mirzoev and Green, 2017). Studies also increasingly examine specific steps, e.g. priority-setting (Maluka *et al.*, 2010, 2011; Barasa *et al.*, 2015). Although there is an increasing knowledge on general planning within urban contexts including for healthy urban environments (Rydin *et al.*, 2012; Barton and Grant, 2013; Hurley *et al.*, 2016), only a handful of studies evaluated urban health planning (Nwameme *et al.*, 2018).

Despite much focus on evidence-informed health policymaking (Bosch-Capblanch *et al.*, 2012; El-Jardali *et al.*, 2012; Mirzoev *et al.*, 2013) or general urban planning (Davoudi, 2006; Faludi and Waterhout, 2006; Krizek *et al.*, 2009; Barton and Grant, 2013), studies specifically examining the role of non-research evidence in urban health planning in LMICs are limited. In this article, we report our analyses of data-informed urban health planning from three Asian cities: Dhaka, Hanoi and Pokhara. We address the following question: how do city governments plan their responses to urban health priority issues, and what is the extent of data use in their planning processes? This article should be of interest and relevance to different stakeholders including academics, policy-makers and funders, who are interested in assessing and improving evidence-informed urban planning.

## Methods

We report qualitative results from a larger Surveys for Urban Equity (SUE) study, which aimed to improve the use of household surveys and facilitate data-informed urban health planning in LMICs (Elsey *et al.*, 2018).

We focus on three Asian cities, where the local governments have a clear mandate for primary health care: Dhaka, Hanoi (capitals of Bangladesh and Vietnam, respectively) and Pokhara (one of six metropolitan cities in Nepal). Their choice was driven by:

- our interest to compare and contrast contexts with different resource availability (Hanoi is more affluent),
- different degrees of political and financial autonomy (more fully devolved context of Nepal in early years of federalization with more centrally driven context of Vietnam),
- different organizations of health systems (publicly dominated Vietnamese system with pluralistic system in Bangladesh) and
- different points in their epidemiological transition, with non-communicable diseases (NCDs) accounting for 77% of deaths in Vietnam, 67% in Bangladesh and 66% in Nepal (Van Minh *et al.*, 2017; WHO, 2019).

The focus of our analysis is on the role of data from mainstream government-administered information systems within annual urban plans developed by the city governments at the lowest administrative level (Dhaka City Corporation, Long Bien district health bureau in Hanoi and Pokhara municipality). This is because mainstream government systems provide regular and stable information source and annual plans typically operationalize multiple policies and strategies and guide the actual implementation of activities. Two caveats are appropriate. First, we recognize that other forms of evidence can inform planning decisions, e.g. research (Rychetnik *et al.*, 2002; 2004; Nutley *et al.*, 2012; Mirzoev *et al.*, 2013; Witter *et al.*, 2019). Second, we acknowledge that data (and other forms of evidence) can inform multiple urban policies and plans across the national and local levels. Our primary focus remains on the use of data from mainstream information systems within annual urban plans. However, to help understand the context of urban health planning, in reporting results, we also highlight the range of urban health plans, identify planning actors and explore key influences on data-informed planning across the local and national levels.

**Figure 1.**
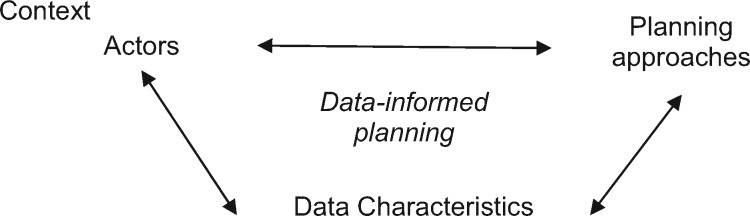
Framework for evidence-informed urban health planning.

Our theoretical framework (see Figure 1), stems from the conceptualizations of evidence-informed policymaking (Mirzoev *et al.*, 2013, 2017), health planning (Green, 2007; Mirzoev and Green, 2017) and the health policy triangle (Walt and Gilson, 1994).

The extent of data-informed urban health planning is determined by four key issues:

(1) key actors (such as health planners, service providers and community representatives) including their roles in generating, disseminating and using data throughout the planning processes;
(2) approaches to and processes of, developing and implementing urban plans;
(3) characteristics of the available data such as timeliness, quality and availability; and
(4) contextual facilitators and constraints to data-informed planning, including but not limited to politics, individual values, societal priorities and traditions, culture of evidence-informed decision-making and availability of resources for generating and disseminating data

The four issues are inter-related. For example, actors’ perceptions of what constitutes robust data for planning decisions shape their decisions as to which data to value, prioritize and eventually use (or not) in their planning decisions. The country’s political environment and governance approaches result in top-down or bottom-up planning approaches, which determine opportunities and processes for using particular data types such as locally available datasets.

Data can be used by the key actors to inform either development or implementation of plans. For example, data can inform situational analyses and prioritization during the plan’s development, whereas during implementation data are required for monitoring and evaluation.

All qualitative data for this study were collected between August 2017 and October 2018 using in-depth interviews (IDIs) with key actors, document reviews and observations (see Table 1).

**Table 1.** Data collection methods

Method	Dhaka	Hanoi	Pokhara	Total
Total IDIs	11	8	13	32
National level actors (relevant ministry, Statistics Office)	4	1	1	6
City governments and health facilities	4	7	12	23
International organizations	3			3
Document reviews	8	54	16	78
Observations			2	2

The IDIs were conducted with 32 purposefully identified participants with key roles in urban health policy, planning and data management, to understand their views and experiences. Purposive sampling was conducted by researchers with detailed knowledge of local contexts. The IDIs were guided by a question guide, which followed our framework and included questions on: planning approaches and processes; data systems; actors and their roles; data use; and wider context. Each interview was face-to-face, lasted 45–60 min and was conducted in local languages (Bengali, Nepali and Vietnamese). Following obtaining informed consent, all interviews were audio-recorded, transcribed and translated, coded and then analysed using Framework Approach which allows for pre-determined themes to be supplemented by further additions emerging from data (Ritchie and Spencer, 1994; Gale *et al.*, 2013). The interview transcripts were coded using five information areas from the question guide and further detailed codes were added to summarize sub-themes and specific findings. Data analysis from each city was conducted using primary data uploaded and coded using NVivo version 10, which led to city-specific research reports. The cross-city comparative analysis was conducted using both primary data available in NVivo format from each city and using research reports from each city. A Microsoft Excel table format was used to summarize data from each city by the five information areas. This summary was extensively discussed and peer-reviewed through several face-to-face meetings and email exchange among researchers from all three cities and eventually informed synthesis of cross-city comparative results.

Review of 78 key documents was conducted to understand current institutional and policy environments. These documents included urban health plans and related guidelines, urban health policies and relevant programmes and projects. Documents were identified through searching in the public domain (ministry websites), references from policies and plans, recommendations from IDI participants, and researchers’ knowledge. We also examined the extent to which health was addressed within non-health urban plans. A semi-structured proforma was used to extract relevant information from each document. It included two sections: (1) basic information about the document and its source and (2) key issues related to each of five information areas (i.e. planning approaches and processes; data systems; actors and their roles; data use; and wider context). The information from completed proformas was used to triangulate results from the IDIs.

Non-participant observations of key urban health planning events (e.g. bi-annual and annual reviews) were planned to allow better understanding of the extent of data-informed planning. However, only researchers in Pokhara were able to gain access to, and eventually observed, a total of two such events. Similar to document reviews, the original intention was to document results in a semi-structured proforma with two sections (basic information about the event and any reflections on each of five information areas). However, researchers documented their reflections in an unstructured format as it allowed for more flexible reflections on processes of interaction using the event. These unstructured notes were used to triangulate results from the IDIs and documents.

The interview transcripts formed the main dataset which was analysed using Framework Approach. Completed semi-structured proformas from the document reviews and unstructured observation notes were used to triangulate (confirm or refute) emerging results from the analysis of the IDI transcripts. All resulting themes were structured under the five information areas.

Ethics approvals were obtained from the University of Leeds (ref: MREC16-137), Hanoi University of Public Health (ref: 324/2017/YTCC-HD3), Bangladesh Medical Research Council (ref: BMRC/NREC/RP/2016-2019/317) and Nepal Health Research Council (ref: 1761).

## Results

Multiple plans affecting urban health exist within each context (see Table 2). These typically comprise 5-year policies and strategies, operationalized through annual plans within the respective budget cycles.

**Table 2.** Urban health policies and plans in each context

Context		
Bangladesh	Nepal	Vietnam
Fourth Health Sector Strategic Investment Plan (SIP)—2017–21, including its implementation plan National Urban Health Strategy (2014) Urban Primary Health Care Service Delivery Project (UPHCSDP) plan 2017–21 Annual City Corporation plan and budget	National Health Policy Urban Health Policy (2017) Periodic 5-year plan (Nepal Health Sector Strategy) Municipal Health Policy Periodic 5-year municipal plan Annual municipal workplan and budget	10-year Socio-Economic Development Strategy, 5-year Socio-Economic Development Strategy National Strategy to Protect, Care, and Improve People’s Health for 2011–20 10- and 5-year health sectoral plans Annual general health plan, multiple vertical health programme plans (*n* = 43)

Both Bangladeshi and Nepali health systems have distinct urban health policies (National Urban Health strategy 2014 and Urban Health Policy 2017, respectively), whereas in Vietnam urban health is addressed through a policy framework for socio-economic development which originates outside the health sector and progresses from a Communist Party’s resolution. In each context, urban health issues are primarily addressed through multiple vertical health programmes and more general city plans, i.e. city corporations in Dhaka, municipality in Pokhara and city and district government in Hanoi. However, health issues are also included within other sectors’ policies and plans in Dhaka (e.g. waste management, sanitation and portable water supply), Pokhara (e.g. road development and drinking water) and Hanoi (e.g. population development policy agenda and health insurance).

Health is one of many competing priorities within each city. Although this clearly reflects the city governments’ multi-sectoral mandate, as one respondent reflected:

*health [is] not the first priority for the Local Government or the City Corporation, rather, their number one priority is roads, and then the drainage system, mosquito control* (national planner, Dhaka).

Next, we present the results by the components of our framework, i.e. planning actors, approaches and processes, data and its characteristics, extent of data use, and key influences on data-informed planning.

### Key planning actors and structures

Urban health planning involves actors across the national, province and local levels (see Table 3). The pluralistic health system in Bangladesh has separate structures for urban and rural health, in Nepal three tiers of government possess similar powers and in Vietnam there is strong system of vertical accountability, as we set out next.

**Table 3.** Key actors in urban health planning in each context

Level	Country		
	Bangladesh	Nepal	Vietnam
National	Ministry of Local Government, Rural Development and Co-operatives Ministry of Health and Family Welfare	Ministry of Health and Population	The Central Committee Ministry of Planning and Investment Ministry of Health
Regional	Dhaka City Corporation (North/South) Urban Primary Health Care Service Delivery Project Smiling Sun (NGO Health Service Delivery Project)	Province government (includes Ministry of Social Development)	Hanoi people’s committee (Include Department of Planning and Investment) Hanoi Health Bureau (includes departments of planning)
District		Pokhara municipality (seven committees including health)	Long Bien People’s Committee (includes health division) Long Bien district health centre (includes department of planning)
Sub-district (including community)		Members of HFOMC Tole/community-level consumer forums, health volunteers, health workers and teachers	Commune health stations

In Bangladesh, the Ministry of Health and Family Welfare (MOHFW) and the Ministry of Local Government, Rural Development and Cooperatives (MOLGRDC) are responsible for rural and urban health, respectively. The pluralistic health system includes a large private sector comprising for-profit (private hospitals, clinics, pharmacies) and not-for-profit [non-governmental organizations (NGOs), traditional practitioners] sectors with both experiencing substantial influence of international organizations such as DFID and The World Bank, through the health Sector Wide Approach. Local governments in urban areas are single-tier, whereas rural local governments include three tiers (Zila/district, Upazila/sub-district and union Parishads). Smaller cities have municipalities, whereas in Dhaka there are two City Corporations: North and South. These are autonomous bodies headed by an elected Mayor who approves administrative and financial matters and chairs Councillor’s meetings. The chief executive officer of a city corporation is appointed by the Government, reports to the Mayor and is the executive Head of the CC and monitors all departmental activities. Health is amongst 11 departments in both municipalities and city corporations and is led by the Chief Health Officer who in Dhaka oversees five zonal offices and works with 56 Ward Councillors. Urban health service provision follows a project-based approach and includes successive 5-year Urban Primary Health Care Service Delivery Projects (UPHCSDP) and The Bangladesh Smiling Sun Franchise Program (BSSFP). The UPHCSDP is a Public–Private Partnership, implemented by the Health Departments of the City Corporations and selected Municipalities, with the financial support of Asian Development Bank, Swedish International Development Cooperation Agency and the United Nations Population Fund. The BSSFP is funded by a USAID/Bangladesh to provide essential healthcare through local NGOs.

In Nepal, recently implemented political devolution has resulted in three tiers of government with similar distribution of powers: Federal, Province and Local. At the Federal level, urban and rural health is a prerogative of the Ministry of Health and Population (MOHP), at the Province level the Ministry of Social Development (MOSD, which covers health and education) and each of 753 Local Governments have Health Units/Section/Division with the mandate to deliver basic health services. Each of three tiers develops its own plans, adapting the format set by the Federal Government to the local context. Federal government sets health budget as conditional grants to sub-national governments, supplemented by provincial and local health budgets. There is a substantial private sector, mostly dominant in urban areas. Influences from the international organizations are visible mostly at the Federal level, e.g. through negotiations between the government and donors (DFID, The World Bank, GAVI and KfW) on resource allocation as part of the Sector Wide Approach. The key urban health planners in Pokhara include the municipality’s Executive Committees and sectoral committees (health section is one of the six sectoral committees) and members of the Health Facility Operational Management Committee (HFOMC) which is headed by an elected Ward chair with the facility incharge (i.e. doctor incharge of managing a health facility) being the member secretary.

In Vietnam, there is a four-tier publicly dominated health system in which the national Ministry of Health retains strong lines of accountability for health-related issues at the Province, District and Commune levels through Province Health Bureaus and District Health Centres. These structures are responsible for both urban and rural health, and the recently implemented decentralization gives greatest autonomy to Province Health Bureaus. The People’s Committees at all levels provide an oversight of, and often funding for, health-related activities (such as preventive medicine) through their health units, but health service delivery is conducted by the health facilities under the Ministry of Health. There is some influence of international organizations though it is not substantial. There is a dedicated evaluation unit within the District People’s Committee in Hanoi.

### Planning processes and approaches

**Figure 2.**
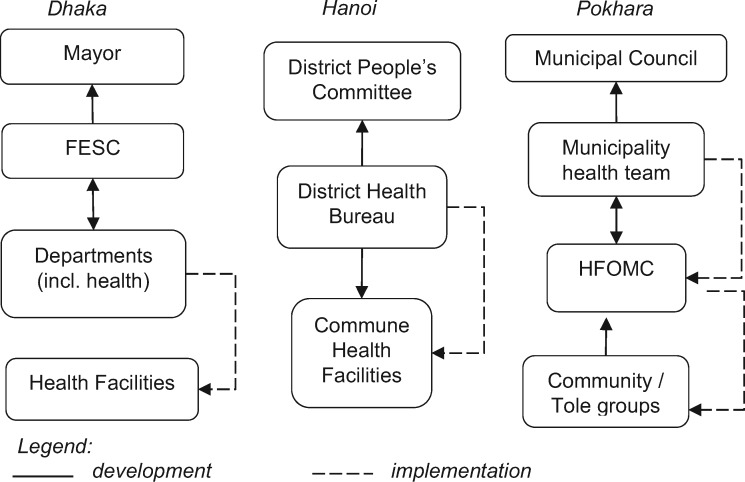
Authors’ visualization of annual planning in Dhaka, Hanoi and Pokhara.

Annual planning in all three cities follows structured processes (see Figure 2), though their different approaches have distinct strengths and limitations. As we explain below, the project-driven planning in Dhaka allows for targeted interventions but leads to fragmentation; in Hanoi the primarily top-down planning allows for greater consistency but leaves little opportunity for local actors’ involvement; and in Pokhara the primarily bottom-up planning approach allows for better identification of local needs but may lead to under-recognition of health as a priority.

Annual planning in Dhaka City Corporations coincides with the government’s July to June financial year and involves steps to primarily co-ordinate budgeting of activities. It starts in mid-March when the Accounts Department of the Mayor’s office issues guidance to all departments—including chief health officer in the health department—for developing the annual budgets. In the following 2 months, the health department consolidates budget projections from all wards. In doing so, the Ward Councillors are expected to engage with community leaders to identify their priority needs, though the degree to which local communities—particularly the urban poor—are engaged in this process was unclear from the data. The councillors then work with Zonal Executive Officers to prepare the budget projections which are then submitted by 15 May to the Financial Establishment Standing Committee (FESC) for review and forwarding for the Mayor’s approval by 30 May and subsequent forwarding to the MOLGRDC, Chief Health Officer and all Ward Councillors.

Dhaka’s pluralistic and project-based urban health system has multiple parallel project-driven planning processes, involving substantial influence of development partners and NGOs:

*when this program is designed […] opinions are exchanged with other Ministries or different stakeholders like NGOs, Civil Society, even donors have done many appraisals […] Here, many donor agencies are providing funding* (national planner, Dhaka).

Hanoi’s mostly top-down annual planning coincides with the December to January financial year. The province-level directive is usually received in August to guide data collection for ‘evaluation of socio-economic development’, which together with the province-level plan inform the primary healthcare (PHC) plan by the department of planning of the District Health Bureau. The PHC plan, after its approval by the district People’s Committee around October, guides development of vertical plans which are approved by the District Health Bureau. Further plans which require funding (e.g. infrastructure development, human resources) are approved by the People’s Committee in December. The annual budgets are usually received around February, meaning that implementation commences from February to March.

The detailed province-level guidance means that cities mostly implement national and provincial priorities with little room for locally identified needs:

*the plan is already formulated by Provincial Health Department… There are no programs or activities in addition* (district health manager, Hanoi).

Furthermore, lengthy processes and strict approval deadlines leave insufficient time for adequate involvement of actors across the different levels:

…*there are a lot of steps and stakeholders need to involve and one month is not enough…* (national health planner, Hanoi).

In Nepal, annual planning aligns with the fiscal year which starts in mid-July and starts with the budgetary ceiling provided by the National Planning Commission along with strategic planning guidelines. In Pokhara, the share of Federal conditional grant is about half of the overall health budget. The process begins with Tole or community-level stakeholder meetings involving consumer forums, health volunteers, health workers and local school teachers at which they identify their priorities. These should then inform meetings in health facilities where the HFOMC develops a detailed health facility plan, adapting the format set out by the Federal Government. These plans are consolidated by the municipality’s health section and after being integrated within the overall municipality development plan, are eventually approved by the Municipal Council and their implementation is monitored through 6-monthly progress reviews.

One decision-maker reflected that integrating the local level with the federal and provincial levels ‘help[s] bring about long-lasting goals in the health care sector, which will help bring development’ (urban planner, Pokhara). However, other participants suggested that the primarily bottom-up approach can lead to under-recognition of health-related issues:

*I have been working with health workers and attending several planning meetings, but I have not found community people come up with health and public health issues… at the tole level* (health staff, Pokhara).

Furthermore, some participants negatively reflected on the lengthy nature of municipality-level planning where the sectoral committee spends over a month collating facility plans.

### Data and its characteristics

All three city governments generate and use predominantly quantitative data. In Hanoi and Pokhara the government Health Management Information System (HMIS) provides the main source of information for planning, alongside periodic surveys and monitoring and evaluation from vertical programmes. In contrast, the HMIS in Bangladesh only covers rural areas, with the exception of data on immunizations and tuberculosis control. The Dhaka City Corporations established an electronic data repository which, according to the participants, does not include multiple uncoordinated surveys conducted by the NGOs, international organizations and project-based monitoring and evaluation data:

*…each donor agency has their own indicators for studies and have their own… system […] However, there are no cohesiveness amongst each of them. Majority of these data are not yet included in our national MIS…* (international organisation, Dhaka).

A major issue in Pokhara relates to unavailability of relevant data for urban health planning, particularly on population groups such as the urban poor and from the private sector. Furthermore, document review revealed that data are mostly collected on government priority programmes such as maternal health and immunizations, whereas important NCDs such as diabetes, cardiovascular issues and mental health appear to be neglected. However, some participants beginning to ‘… feel guilty about not including topics on mental health…’ (facility incharge, Pokhara).

Both interviews and documents revealed that accurate population data are often missing. The resulting denominators for population counts, which are crucial for target setting and allocating resources in planning, are incomplete. For example, in Dhaka planners have been using the 2011 census data incremented by population growth rate and in Pokhara no data on the urban poor and migrants were available. In Dhaka and Hanoi, results of targeted surveys are used to identify to the poorest to aid their access to basic healthcare, though it was unclear whether these contain regularly updated data. In Hanoi, there is a structured poverty assessment process which informs allocation of free government health insurance. However, this system omits the rapidly increasing rural migrants because, as one health planner reflected, these groups have temporary residence registration status and are, therefore, ineligible for free government health insurance.

Data sharing for planning in all three contexts is mostly done at meetings, such as the bi-annual reviews in Pokhara. The participants in Hanoi reported that data are often shared as Microsoft Word or Excel files as email attachments, thus raising concerns about data confidentiality. In Dhaka, different actors can be invited to present data at planning meetings, though this rarely involves graphical data representation.

In both Dhaka and Hanoi, accessing official data requires approvals by the respective authorities (Bangladesh Bureau of Statistics and the General Statistics Office, respectively). Such an approach can provide a useful data quality control mechanism by the relevant authorities. However, we found that it can also exclude non-approved multiple datasets generated by the NGOs and projects in Dhaka from informing planning decisions, and in Hanoi such an approach can even lead to removal of data from the planning process:

*…this year, I forgot to check data and the city did not approve it. So, we need to use official data for planning* (health manager, Hanoi).

Duplication, incompleteness and inconsistencies in data were commonly reported. The participants in Hanoi and Dhaka felt their data were of generally good quality, because of quality control by their statistics authorities.

*I think the accuracy of data is about 95–98%. It is because specialist of the programme is going to check monthly data every quarter… and give feedback* (health manager, Hanoi).

When probed on what they perceive as robust data, only in Dhaka did the participants identify specific data attributes such as accuracy, timeliness and disaggregation by gender, and emphasized the importance of qualitative data alongside quantitative measures.

Decision-makers are increasingly interested in information technology to reduce fragmentations in data recording, storing and sharing. In Pokhara, the introduction of the District Health Information System (DHIS2) is a key priority supported by the Federal MoHP and international organizations. In Hanoi, health units have different software for specific issues such as HIV or health insurance, and in addressing this, an electronic inter-sectoral System Administration Manager (eSAM) has been piloted since 2017. It includes personal data from all public services (including health) provided at the commune, district and province/city levels. It creates personal health records of the local population, including self-employed and informal workers to inform activities in encouraging the uptake of annual health checks. However, it omits rural migrants and as one respondent reflected:

*[the] software… is unstable, it stopped for a while and we continue the implementation…* (district health centre manager, Hanoi).

As a result of greater role of technology, the aggregated data are increasingly shared electronically in all three cities, though each is yet to have a centralized data repository.

### Role of data in planning

The participants from all three cities had claimed that their urban health plans are generally informed by the data. However, we found a difference between the intended (as per the documents, guidance and participants’ statements) and the actual use of data in planning. For example, in Hanoi only 5 out of 43 plans had clear references to the data which informed that plan.

When probed for specific examples, clearer experiences were described in Pokhara where the participants specifically highlighted the use of data during HFOMC meetings and bi-annual reviews:

*We look at data of the last two fiscal years as this helps to not only see the recent trend and the change in the community health scenario but also provides an overview on where the gaps are and how we should fill those gaps to achieve the targets and set out further plans* (facility incharge, Pokhara).

The mechanisms for data use in planning were especially unclear in Dhaka, where the participants attributed these due to fragmented government structures and multiple uncoordinated plans. In Hanoi, in addition to the use of HMIS data, examples of data-informed planning also included use of monitoring and evaluation data within vertical programmes:

*[from data] we divide community into three different groups: excellent, good, and medium… for quarterly … bi-monthly and … monthly [support, respectively]* (health planner, Hanoi).

During the interviews, participants reflected on how to improve data-informed planning. In all three cities, there was a clear preference for the integrated electronic data repository to be easily accessible by all planners. This would resolve data duplication and constrained access in Dhaka and Hanoi. In Dhaka, there was a clear preference for greater co-ordination including with the private sector. Although not explicitly mentioned, the time-limited nature of project plans raises a possible need for adequate documentation of learning from these projects and to maintain longer-term institutional memory.

Participants from Hanoi and Pokhara did not identify specific improvements, perhaps reflecting the constrained nature of the top-down planning approach in Vietnam and lack of clarity on the roles and responsibilities due to ongoing decentralization in Nepal.

### Key facilitators and constraints to data-informed planning

We found two groups of common influences on data-informed urban health planning: shortages of human and financial resources, and limitations of current planning approaches. Key context-specific barriers to data-informed planning included fragmented data in Dhaka, the top-down urban health planning in Hanoi, and unavailable, incomplete and inaccurate data in Pokhara. Organizational and system-level determinants of data use emerged clearer in Hanoi and Pokhara, and more specific barriers were identified in Dhaka.

Availability of resources has emerged as a key influence particularly in Dhaka and Pokhara. This included lack of dedicated staff in local governments for data analysis and their limited expertise, and limited resources for data collection and analysis—all leading to unavailable and poor-quality data. All these, along with unclear guidelines constrain data use as one participant reflected:

*There should be clarity on recording and reporting… we can put fever, headache wherever we want … this leads to confusion and does not clearly show the trend or prevalence of health problems …* (health worker, Pokhara).

Furthermore, lack of dedicated resources also constrained planning processes:

*… health related planning is not their priority. They do not have any reasonable budget for health planning* (development partner, Dhaka).

Participants in Dhaka highlighted that national ministries possess technical capacity but this expertise does not trickle down to the City Corporations.

The approach to planning can determine the extent of data use. For example, in Hanoi the top-down planning constrains the identification of local priorities:

*The plan is already formulated by Provincial Health Department and we only change our data to fit with the plan* (health planner, Hanoi).

Reliance on government-approved data in Dhaka and Hanoi can limit the choice of, and access to, data. In contrast, the 6-monthly progress reviews in Pokhara, identified through document reviews, appear to provide a useful structure and process for using data in implementation monitoring, though it is constrained by the non-availability of private sector data.

In Dhaka, a major barrier relates to poorly co-ordinated processes of collecting, analysing and using data including a clear disconnect from the planning processes:

*…there is data coordination problem, so, ‘who will collect data’, ‘who will manage it’—this is the situation of ‘blaming each other’* (national decision-maker, Dhaka).

The resultant existence of multiple datasets in Dhaka contributes to lack of clarity amongst urban health planners regarding the scope and reliability of these different datasets. Other context-specific barriers to data-informed planning emphasized included high staff turnover, absence of urban HMIS, lack of data from the large private sector and international organizations, misaligned data and planning processes, and limited management training of appointed Chief Health Officers.

## Discussion

In this article, we examined the extent of data-driven urban health planning in three Asian cities. Multiple plans are produced within each context. Planning followed primarily top-down approach in Vietnam, included greater community involvement in Nepal and was fragmented in Bangladesh. Plans were claimed to be informed by data from mainstream information systems, though the extent of data-informed planning appears limited. Although the data were often perceived as being of good quality, it provided insufficient detail on the urban poor and migrant populations and was unavailable from the private sector. Key common influences on the data-informed urban health planning included resource shortages (including financial and human resources) and limitations of current planning approaches (e.g. with top-down planning constraining the identification of local priorities and even leading towards retro-fitting data to the nationally set plans in Vietnam).

Empirical studies examined health planning from different perspectives, such as planning for PHC within the decentralized contexts of Nigeria (Eboreime *et al.*, 2018), alignment between operational planning and budgeting and priority-setting at county hospitals in Kenya (Tsofa *et al.*, 2016; Barasa *et al.*, 2017) or using a theoretical lens to draw links between evidence-based practice and evidence-based urban policy and planning decisions (Krizek *et al.*, 2009). We add to, and extend, this growing though still limited body of knowledge through focusing on the extent of data use by the city governments in their urban planning.

City governments can act as hubs for multi-sectoral action. Our results on the position of urban health policies within wider urban policy frameworks highlight the tensions this brings where addressing health-related issues is a competing priority of city governments. A key arising question is whether health being a competing priority prevents addressing health-related issues in a comprehensive manner with dedicated resources, or whether multiple social determinants of health are best addressed in an integrated way. Barton and Grant suggest three levels of such integration. They identified the first level as comprising basic considerations of health within urban planning, the second as mostly addressing the ‘downstream’ implications of urban environmental planning and the third, and rarest, is the fully fledged mutually reinforcing integration of health within urban planning (Barton and Grant, 2013). Urban health planning in Dhaka, Hanoi and Pokhara reflects the first and second levels of integration, thereby highlighting the value of combining the targeted and integrated approaches to address urban health issues—and potentially challenging Barton and Grant’s arguments for the complete integration of health within urban planning.

It is important that urban planners are able to effectively target the needs of the most vulnerable such as the urban poor and migrant populations. For various reasons, these groups are omitted from official statistics thus raising questions about reliability of population denominators and effectiveness of resultant planned activities in relation to reaching the most vulnerable groups. This echoes the current emphases of limited available and accurate data on urban health challenges (Elsey *et al.*, 2016; Wickremasinghe *et al.*, 2016; Shawar and Crane, 2017). Where the data does exist, it does not appear to be effectively disseminated and used. For example, in Dhaka, we found that graphical representation of data is scarce and we did not find evidence of further means of effective data sharing such as through dashboards or press-releases. With the increased focus on health equity and the leaving no-one behind agenda (Pannarunothai *et al.*, 2004; WHO, 2010, 2013; Ottersen *et al.*, 2014; Onoka *et al.*, 2015), a key argument from our results is that data highlighting the needs of the most marginalized urban poor and migrant populations should be available within existing information systems.

Perhaps unsurprisingly, the urban planners did not conceptually demarcate detailed stages in their planning processes such as situational analysis, priority-setting and option appraisal (Green, 2007), even though these were included in the guidance documents such as the MOHP’s planning and budgeting guidelines in Nepal (MoHP, 2018). Instead, the participants had just highlighted development and implementation. Such an approach is more pragmatic and is similar to two-stage frameworks (Mirzoev and Green, 2015; Wickremasinghe *et al.*, 2016). However, a possible shortcoming of such an approach is that it may identify less windows of opportunity for data use throughout the planning process, e.g. in appraisal of different alternatives during option appraisal. The lack of a distinctly identifiable situational analysis stage also suggests that plans may not always target the rapidly changing disease patterns and risk factors within urban contexts.

The disconnect between planning and data processes which was particularly evident in Dhaka is similar to what was also found in relation to policy processes (i.e. formulation, implementation and evaluation) and evidence (i.e. generation, dissemination and use) processes in other LMICs including Vietnam ( Mirzoev *et al.*, 2013, 2017). This finding may also explain the lack of available data at the ‘correct’ planning stage which was particularly emphasized in Pokhara. Aligning data generation and dissemination with relevant planning steps (such as situational analysis, priority-setting, option appraisal and monitoring and evaluation) may help identify clear windows of opportunity for enhancing evidence-informed urban health planning.

We found that in all three cities planning largely followed the annual budgeting cycle. This finding contrasts with a disconnect found between planning and budgeting in a study from Kenya (Tsofa *et al.*, 2016). Perhaps our results reflect the nature of annual planning as being more closely aligned with budgetary commitments and resource spending on day-to-day basis. On the other hand, the longer-term strategic plans and policies may not always carry firm financial commitments and, therefore, may not require clear links with budgets.

The reliance on government-approved data which we found in Vietnam and Bangladesh can be seen as a strength, allowing for consistent use of available mainstrem datasets. However, it has a potential to exclude other datasets—such as multiple surveys often done by the NGOs and different universities in Bangladesh. Furthermore, bureaucratic delays to data access in Vietnam may constrain the planners’ desire to use data in their decisions. It would be unrealistic to expect the national statistics agencies to change their rules. Instead, the more feasible policy options may include seeking faster approvals of survey data in Bangladesh and reducing the bureaucracy to improve efficiency of data access in Vietnam. The increased interest in information technology in all three cities represents a possible platform for addressing these issues, e.g. through electronic data repositories where the real-time approved data can be easily accessible.

The lack of clear references to the role of local communities in urban health planning processes in Hanoi and Dhaka suggests that their roles are limited in these two contexts. On the other hand, we found that in Pokhara urban health planning does include consultation with community representatives. Different empirical studies highlighted the importance of involving communities in health planning within decentralized contexts of India, Nigeria and Tanzania (Wickremasinghe *et al.*, 2016; Eboreime *et al.*, 2018; Shukla *et al.*, 2018). Our results suggest that enhancing the role of communities, particularly the most vulnerable should help urban health planning become more responsive to the needs of marginalized urban poor and migrant populations.

Different key influences on the use of data in urban health planning were reported from each context, many echoing the existing literature which highlights problems with access and presentation of data from surveys and other sources (Elsey *et al.*, 2016), limited staff expertise to use evidence (Witter *et al.*, 2019), financial constraints (Wickremasinghe *et al.*, 2016) and long-standing dominance of rural health in the development agenda (Shawar and Crane, 2017). Although not emphasized by the participants from Hanoi, potential pressure to achieve the national targets may form a bias towards reporting inaccurately positive results. A substantial presence of international organizations in Bangladesh and Nepal could be a way of bridging resource gaps, e.g. related to staff expertise, though our analysis shows that in Dhaka this contributes to fragmentation, and in Nepal international agencies are not particularly active at the local level.

Key influences on the data-informed planning mostly reflected the meso (organizational) and the macro (systems) level issues. In contrast, the micro (individual) level issues, such as personal values, interests and agendas have not featured prominently in our study. Yet, these can be equally important for evidence-informed decisions, e.g. through shaping the culture of evidence-informed decision-making (Hutchinson *et al.*, 2011; Mirzoev *et al.*, 2017). Absence of references to the micro-level influences may reflect our interviewing approach and the nature of urban planners, as both being more outwards-looking and less self-reflective. This absence may also relate to the relatively limited number of attributes of robust data by the key stakeholders, an area which is worth exploring more in further studies.

On a methodological note, our analysis was guided by a framework for evidence-informed urban health planning which distinguished four key inter-related components: actors, planning approaches, data characteristics and wider context. The presentation of study results has largely followed the individual components of our framework. However, the inter-relationships among the four components also became evident. Examples of such inter-relationships include: (1) implications of omissions of key population groups from the existing datasets on effectiveness of planning decisions to reach the most vulnerable, (2) actors’ conceptualizations of the planning processes and perceptions of what constitutes robust evidence, all informing the extent of data-informed urban health planning and (3) implications of resource shortages, limited expertise and other contextual influences on the generation and utilization of relevant evidence in health planning decisions. On reflection, even though the framework may appear descriptive at first glance, understanding the links between the different components of this framework can contribute to in-depth analysis of evidence-informed nature of urban health planning.

Four policy implications for enhancing evidence-informed urban health planning emerge from our results. First, improving availability and quality of data particularly on urban poor and migrants from both the public and private sectors, should improve targeted planning responses to urban health challenges. Second, aligning different planning processes and introducing specific mechanisms for data use at different planning stages should improve the likelihood of evidence-informed planning. Third, enhancing the role of information technology should help address data fragmentation and improve access to the available datasets. Last but not least, enhancing the involvement of all population groups, particularly marginalized and vulnerable local communities, in planning processes should contribute to participatory decision-making and make planning more responsive to local needs. Of course, any improvements need to recognize, and leverage, the current systemic strengths—such as better alignment and co-ordination within Vietnam’s top-down planning systems, more locally responsive planning and greater community involvement in Nepal’s bottom-up planning systems, and large amount of available expertise within the Bangladeshi pluralistic health system.

### Study limitations

We acknowledge three limitations. First, our focus on one city within each country did not allow for intra-country comparisons. Hanoi and Dhaka are capitals and perhaps atypical examples with greater proximity to national decision-making. Second, we were driven by the participants’ perceptions and we did not ourselves evaluate the actual data use or data quality. We minimized this through triangulation of interview data with observations and document reviews. Last, we had examined role of data within annual urban plans only. Although we believe our paper already adds to the limited literature, future studies may be appropriate to advance the knowledge on this subject through comparing participant views with researcher assessments of data quality and comparing the role of different types of evidence within multiple plans across different sectors.

## Conclusions

City governments are well-positioned to effectively address urban health issues with their inter-sectoral mandate. Urban health planning, which underpins effective responses to urban health issues, requires good quality and timely data. Although multiple systems produce health-related data, its role in planning appears limited. Key common influences on data-informed planning include constrained resources and limitations of current planning approaches. Feasible improvements to evidence-informed urban health planning include improving availability and quality of data particularly on urban poor and migrants, aligning different planning processes and introducing clearer mechanisms for data use, enhancing the role of information technology and improving the involvement of communities in urban health planning processes.
